# Efficacy and safety of tirofiban for acute ischemic stroke without large and medium vessel occlusion: a systematic review and meta-analysis

**DOI:** 10.3389/fneur.2026.1823316

**Published:** 2026-05-04

**Authors:** Jiaqi Jiao, Jiawei Zhang, Xuehui Lan, Junhong Guo, Shaoshuai Wang

**Affiliations:** Department of Neurology, The First Hospital of Shanxi Medical University, Taiyuan, China

**Keywords:** acute ischemic stroke, functional outcome, meta-analysis, tirofiban, vessel occlusion

## Abstract

**Background:**

Early and effective intervention is crucial in the management of acute ischemic stroke (AIS) without large- or medium-vessel occlusion (non-LVO/MVO), which accounts for approximately 60–70% of all AIS cases. Tirofiban has been investigated as a therapeutic option for non-LVO/MVO AIS. However, existing studies have reported inconsistent findings regarding its efficacy and safety, and high-level evidence derived from large-scale pooled analyses remains lacking.

**Aims:**

This study aimed to conduct a systematic review and meta-analysis to assess the efficacy and safety of tirofiban in patients with non-LVO/MVO AIS, and to further explore the influence of intravenous thrombolysis (IVT) status and study design on treatment outcomes.

**Summary of review:**

PubMed, Web of Science, Embase, and the Cochrane Library were systematically searched from inception to December 2025. Eligible studies included patients with non-LVO/MVO AIS, compared tirofiban with conventional antiplatelet therapy, and reported original data on functional or safety outcomes. The primary efficacy outcomes were excellent functional outcome at 90 days (modified Rankin Scale [mRS] score 0–1) and favorable functional outcome (mRS score 0–2). Safety outcomes included symptomatic intracerebral hemorrhage (sICH), 90-day mortality, and peripheral bleeding. Subgroup analyses were conducted according to IVT status and study design. A total of 1,678 records were identified, of which nine studies met the inclusion criteria, encompassing 3,225 patients. Tirofiban was associated with a significantly higher likelihood of achieving a 90-day excellent functional outcome (odds ratio [OR] 1.66, 95% confidence interval [CI] 1.34–2.06; *p* < 0.001; *I^2^* = 26%) and favorable functional outcome (OR 1.79, 95% CI 1.30–2.47; *p* < 0.001; *I^2^* = 58%). Regarding safety, tirofiban did not significantly increase the risk of sICH (OR 4.02, 95% CI 0.91–17.70; *p* = 0.07; *I^2^* = 5%) or 90-day mortality (OR 1.06, 95% CI 0.53–2.12; *p* = 0.87; *I^2^* = 37%). However, it was associated with a significantly higher risk of peripheral bleeding (OR 1.87, 95% CI 1.32–2.66; *p* < 0.001; *I^2^* = 0%). Subgroup analyses demonstrated tirofiban conferred significant functional benefits exclusively in non-IVT patients, whereas no such improvement was observed in patients with prior IVT. However, particularly robust and homogeneous effects observed in the randomized controlled trial (RCT) subgroup (*I^2^* = 0% for mRS 0–1).

**Conclusion:**

Tirofiban significantly improves 90-day functional outcomes in patients with non-LVO/MVO acute ischemic stroke, with primarily observed in patients without prior IVT. The clinical utility of adding tirofiban post-IVT remains unproven. Although its use was not associated with an increased risk of severe bleeding or mortality, the higher incidence of peripheral bleeding warrants careful monitoring in clinical practice.

## 1 Introduction

AIS remains a major global health burden. It is among the leading causes of death worldwide, ranks third in terms of death and disability measured by disability-adjusted life years (DALYs), and is a significant contributor to dementia (1). Early restoration of cerebral perfusion is currently the only proven effective therapeutic strategy. Intravenous thrombolysis (IVT) is the first-line reperfusion therapy for eligible patients within 4.5 h of symptom onset; however, its use is restricted by a narrow therapeutic window, an increased risk of hemorrhage, and potential neurotoxic effects (2). In recent years, advances in endovascular therapy (EVT) have extended the reperfusion window, but mechanical thrombectomy (MT) is primarily indicated for patients with large vessel occlusion (LVO) (3).

For the majority of patients with non-LVO/MVO AIS most commonly those with small vessel occlusion or branch atherosclerotic disease (BAD) treatment options remain limited. Many present beyond the IVT time window, have contraindications to thrombolysis, or are not candidates for EVT. Consequently, management largely relies on conventional oral antiplatelet agents, such as aspirin and clopidogrel. However, these agents have a delayed onset of action and may inadequately inhibit ongoing microthrombus formation in certain patients, contributing to END, which occurs in approximately 5–40% of cases and is associated with poor long-term functional outcomes (4). Therefore, identifying more effective acute therapeutic strategies to enhance neurological recovery and improve prognosis in this population represents an urgent clinical need.

Tirofiban is a highly selective and rapidly acting platelet glycoprotein IIb/IIIa receptor antagonist that directly inhibits the final common pathway of platelet aggregation, thereby effectively preventing thrombus formation and propagation. It is characterized by a rapid onset of action, a short half-life (approximately 1.5–2 h), and prompt recovery of platelet function after discontinuation, making it particularly suitable for short-term antithrombotic therapy in clinical settings with an elevated bleeding risk (5). In addition to its antiplatelet effects, experimental evidence suggests that tirofiban may exert neuroprotective properties in acute AIS by modulating microglial polarization and attenuating oxidative stress, thereby mitigating inflammatory responses (6, 7). Clinically, tirofiban has been applied in early-stage AIS management, as rescue therapy following unsuccessful EVT, and in combination with thrombolytic agents (8), with accumulating evidence supporting its potential role in preventing stroke progression.

In recent years, several clinical studies have investigated the efficacy and safety of tirofiban in patients with non-LVO/MVO AIS; however, the findings remain inconsistent. Some studies have reported that tirofiban significantly alleviates neurological deficits (9), and a recent meta-analysis suggested potential benefits among patients who did not receive reperfusion therapy (10). Conversely, other reports have raised concerns regarding a possible increased risk of intracranial hemorrhage associated with tirofiban use (11). To date, high-quality evidence derived from large-scale pooled analyses remains insufficient to definitively inform its clinical application.

Therefore, this systematic review and meta-analysis was conducted to comprehensively assess the efficacy and safety of tirofiban in patients with non-LVO/MVO acute ischemic stroke through the systematic inclusion of relevant clinical studies, with particular emphasis on functional and safety outcomes. The objective was to clarify its clinical risk–benefit profile and to provide robust evidence to inform therapeutic decision-making in this patient population.

## 2 Methods

### 2.1 Search strategy

This meta-analysis was conducted in accordance with the Preferred Reporting Items for Systematic Reviews and Meta-Analyses (PRISMA) guidelines (12). PubMed, Embase, the Cochrane Library, and Web of Science were systematically searched for English-language studies published from database inception through December 2025. The search strategy combined Medical Subject Headings (MeSH) and free-text terms as follows: (“stroke” OR AIS OR “cerebral infarction” OR “cerebral ischemia” OR “brain infarction” OR “lacunar infarction”) AND “tirofiban.” Randomized controlled trials (RCTs) and observational studies comparing tirofiban with non-tirofiban therapy in patients with non-LVO/MVO AIS were eligible for inclusion. All retrieved records were imported into EndNote 21, and two reviewers independently screened titles and abstracts according to predefined inclusion and exclusion criteria. This meta-analysis was prospectively registered in PROSPERO (ID: CRD420251276430). No substantial deviations from the prespecified protocol occurred during the review process.

### 2.2 Data collection and quality assessment

Inclusion criteria: (1) RCTs, prospective cohort studies, or retrospective case–control studies published in English; (2) patients diagnosed with non-LVO/MVO acute ischemic stroke; (3) comparison of the efficacy and safety of tirofiban versus a control group; and (4) reporting of at least one of the following outcomes: safety outcomes (any intracerebral hemorrhage, symptomatic intracerebral hemorrhage [sICH], peripheral bleeding, or 90-day mortality) or efficacy outcomes (90-day modified Rankin Scale [mRS] score). Exclusion criteria: (1) reviews, case reports, conference abstracts, animal studies, or duplicate publications; and (2) studies with incomplete data or from which relevant data could not be extracted.

Quality assessment was conducted using Review Manager 5.4.1. The Cochrane Risk of Bias 2 (ROB 2) tool was applied to RCTs (13), and the Risk Of Bias In Non-randomised Studies-of Interventions (BOBINS-I) was used to evaluate retrospective observational studies (14).

### 2.3 Outcome measures

Two reviewers independently extracted data using a predesigned form, with any disagreements resolved through discussion with a third specialist. Extracted information included the author, publication year, study design, sample size, patient age, IVT status, and tirofiban dosage and administration route. The primary efficacy outcomes were 90-day excellent functional outcome, defined as a modified Rankin Scale (mRS) score of 0–1, and favorable functional outcome (mRS 0–2). Safety outcomes included 90-day intracerebral hemorrhage (any ICH), symptomatic intracerebral hemorrhage (sICH), 90-day mortality, and peripheral bleeding.

### 2.4 Statistical analysis

Statistical analyses were performed using Review Manager 5.4, with a significance threshold set at *p* < 0.05. Categorical outcomes were expressed as odds ratios (ORs) with 95% confidence intervals (CIs), while continuous outcomes were presented as mean differences (MDs) with 95% CIs. Statistical heterogeneity across studies was assessed using the *I^2^* statistic and classified as low (*I^2^* < 25%), moderate (25% ≤ *I^2^* < 50%), or high (*I^2^* ≥ 50%). A random-effects model was applied to account for potential heterogeneity among studies. To explore potential sources of heterogeneity and evaluate the impact of clinical variables, subgroup analyses were conducted based on IVT status and study design. Sensitivity analyses were performed using the leave-one-out method to assess the robustness of the pooled results. Publication bias was visually evaluated with funnel plots.

## 3 Results

### 3.1 Search results

**Figure 1:**
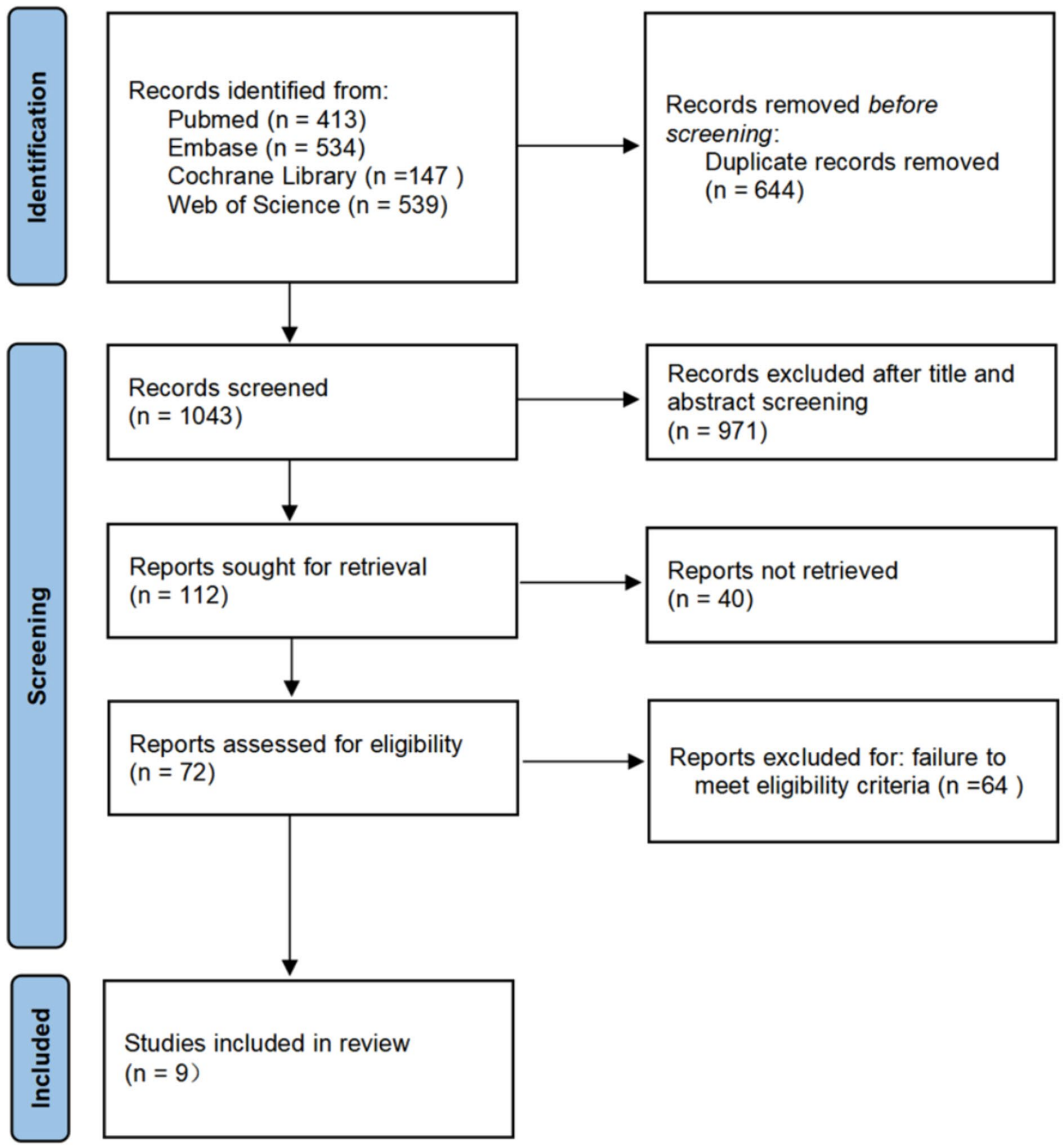
PRISMA (preferred reporting items for systematic reviews and meta-analyses) flowchart for screening.

A total of 1,678 records were identified (PubMed: 413; Embase: 534; Cochrane Library: 147; Web of Science: 593). After removing duplicates in EndNote 21, 1,043 records remained. Following title and abstract screening, 72 articles were selected for full-text review. Ultimately, nine studies met the inclusion criteria, comprising six RCTs and three observational studies (including one post-hoc analysis of an RCT). The study selection process is illustrated in the PRISMA flowchart (Figure 1).

### 3.2 Study characteristics

The nine included studies were published between 2017 and 2025, encompassing a total of 3,225 patients (1,644 in the tirofiban group and 1,581 in the control group) (15–23). All studies compared tirofiban with other antiplatelet agents, such as aspirin or clopidogrel. One study administered tirofiban intra-arterially, while the remaining studies used intravenous administration. Four studies included patients who had received IVT, and one study specifically enrolled patients who experienced functional deterioration after IVT. Most studies administered tirofiban at a dose of 0.4 μg/(kg·min) via intravenous infusion for 30 min, followed by a maintenance dose of 0.1 μg/kg/min for 24 to 48 h. Detailed characteristics of the included studies are presented in Tables 1, 2.

**Table 1:** Study characteristics.

Study ID	Study period	Study design	Sample size (experimental/control)	Thrombolysis administered	Administration route	Dosage	Duration	Main inclusion criteria	Main exclusion criteria
Han et al. (2022) (15)	June 2019–December 2020	RCT, double-blind	177/180	No	IV	Loading: 0.4 μg/kg/min for 30 min Maintenance: 0.1 μg/kg/min	24–48 h	1. Acute ischemic stroke (AIS) without large/medium vessel occlusion; 2. Onset within 12 h; 3. NIHSS score ≥4; 4. No cardioembolic etiology	1. Large/medium vessel occlusion; 2. Cardioembolic stroke; 3. Intracranial hemorrhage; 4. Platelet count <100 × 10^9^/L
Liu et al. (2020) (16)	June 2017–May 2022	Prospective, Randomized Pilot Study, Open-label	10/7	Yes (Urokinase)	IV	Loading: 0.4 μg/kg/min for 30 min Maintenance: 0.1 μg/kg/min	48 h	1. Branch atheromatous disease (BAD) with early neurological deterioration (END) post-intravenous thrombolysis (IVT); 2. Onset <6 h; 3. No intracranial/systemic bleeding post-IVT	1. Intracranial hemorrhage/malignant edema; 2. Cardioembolic stroke (e.g., atrial fibrillation); 3. Thrombocytopenia (≤100 × 10^9^/L); 4. Severe organ insufficiency
Wang et al. (2025) (17)	Not specified	Retrospective, Subgroup Analysis (TREND Trial)	117/105	No	IV	Loading: 0.4 μg/kg/min for 30 min Maintenance: 0.1 μg/kg/min	24–72 h	1. AIS without large/medium vessel occlusion; 2. Onset within 24 h; 3. Stratified by intracranial artery stenosis degree	1. Large/medium vessel occlusion; 2. Cardioembolic stroke; 3. Intracranial hemorrhage; 4. Severe renal/hepatic insufficiency
Lin et al. (2017) (18)	January 2016–September 2016	Prospective, Single-arm Study with Historical Cohort	25/25	No	IV	Loading: 0.4 μg/kg/min for 30 min Maintenance: 0.1 μg/kg/min	24 h	1. AIS without arterial occlusion on neurovascular imaging; 2. Onset 4.5–24 h; 3. NIHSS score ≥4; 4. Out of thrombolytic window	1. Arterial occlusion; 2. Intracranial hemorrhage; 3. Coagulopathy/anticoagulant use (INR > 1.7); 4. Platelet count <100 × 10^9^/L
Zi et al. (2023) (19)	October 2020–June 2022	Multicenter, Prospective, RCT, Double-blind, Double-dummy	606/571	Yes (for post-IVT END)	IV	Loading: 0.4 μg/kg/min for 30 min Maintenance: 0.1 μg/kg/min	48 h	1. AIS without large/medium vessel occlusion; 2. NIHSS score ≥5 with moderate/severe limb weakness; 3. Onset ≤24 h (or symptom progression 24–96 h, or END/no improvement post-IVT)	1. Large/medium vessel occlusion; 2. Cardioembolic stroke; 3. Intracranial hemorrhage; 4. Severe bleeding tendency
Zhang et al. (2022) (20)	June 2018–May 2022	Single-center, Prospective, Randomized Pilot Study, Open-label	59/14	Yes (rt-PA)	IV	Loading: 0.4 μg/kg/min for 30 min Maintenance: 0.1 μg/kg/min	24 h	1. AIS with END post-IVT (NIHSS increase ≥4); 2. Onset <4.5 h; 3. No intracranial hemorrhage post-IVT	1. Large/medium vessel occlusion; 2. Cardioembolic stroke; 3. Intracranial hemorrhage/malignant edema; 4. Thrombocytopenia (≤100 × 10^9^/L)
Xue et al. (2025) (21)	July 2021–January 2023	Single-center, Retrospective, Case-control Study	128/102	No	IA + IV	Intra-arterial (IA): 0.4–0.5 mg (8–10 mL) bolus Maintenance (IV): 0.1 μg/kg/min	24 h	1. AIS without large/medium vessel occlusion; 2. Onset 6–24 h (out of thrombolytic window); 3. No intracranial hemorrhage	1. Large/medium vessel occlusion; 2. IVT/EVT history; 3. Severe organ insufficiency; 4. Bleeding tendency
Yu et al. (2022) (22)	Not specified	Single-center, Prospective, RCT, Open-label	134/133	No	IV	Loading: 0.4 μg/kg/min for 30 min Maintenance: 0.1 μg/kg/min	72–108 h	1. AIS without large-vessel occlusion; 2. Onset ≤72 h; 3. NIHSS score <20; 4. Out of thrombolytic window or ineligible for IVT	1. Large-vessel occlusion requiring EVT; 2. IVT history; 3. Intracranial hemorrhage; 4. Pre-stroke mRS score ≥2; 5. Severe coagulopathy
Tao et al. (2025) (23)	September 2022–March 2024	Multicenter, Phase III, RCT, double-blind	414/418	Yes (alteplase/tenecteplase)	IV	Loading: 0.4 μg/kg/min for 30 min Maintenance: 0.1 μg/kg/min	24 h	1. Acute non-cardioembolic AIS; 2. IVT (alteplase/tenecteplase) within 4.5 h of onset; 3. Age ≥18 years	1. Large vessel occlusion requiring EVT; 2. Intracranial hemorrhage on baseline imaging; 3. Severe bleeding tendency; 4. Severe renal/hepatic dysfunction; 5. Pregnancy/lactation

**Table 2:** Baseline data.

Study ID	Age		Gender (male)		Hypertension (*n*/%)		Diabetes (*n*/%)		Baseline NIHSS score	
	Tirofiban	Control	Tirofiban	Control	Tirofiban	Control	Tirofiban	Control	Tirofiban	Control
Han et al. (2022) (15)	67 (59–75)	67 (59–74)	86 (48.6%)	78 (43.3%)	110 (62.1%)	100 (55.6%)	61 (34.5%)	52 (28.9%)	6 (4–8)	5 (4–8)
Liu et al. (2020) (16)	58.50 ± 9.03	64.71 ± 10.48	7 (70.0%)	5 (71.4%)	6 (60.0%)	4 (57.1%)	3 (30.0%)	2 (28.6%)	7.00 (4.00,9.25)	7.19 (5.00,9.00)
Wang et al. (2025) (17)	62.67 ± 9.72	67 (63.8%) (43 + 24)	76 (65.0%)	68 (64.8%)	79 (67.5%)	69 (65.7%)	42 (35.9%)	36 (34.3%)	5.00 [4.00–6.50]	5.00 [4.00–6.00]
Lin et al. (2017) (18)	63 (47–82)	70 (44–89)	16 (64.0%)	15 (60.0%)	17 (68.0%)	16 (64.0%)	9 (36.0%)	8 (32.0%)	6 (4–15)	7 (4–13)
Zi et al. (2023) (19)	68.0 (58–75)	68.0 (59–76)	372 (61.4%)	348 (61.0%)	400 (66.0%)	375 (65.7%)	212 (35.0%)	195 (34.1%)	9.0 (7.0–10.0)	9.0 (7.0–10.0)
Zhang et al. (2022) (20)	69.24 ± 14.88	68.21 ± 12.86	38 (64.4%)	9 (64.3%)	40 (67.8%)	9 (64.3%)	21 (35.6%)	5 (35.7%)	8.90 ± 2.75	8.14 ± 3.51
Xue et al. (2025) (21)	62.57 ± 9.14	62.63 ± 10.32	91 (71.1%)	66 (64.7%)	73 (57.0%)	55 (53.9%)	26 (20.3%)	30 (29.4%)	5.00 [3.00,8.00]	5.00 [3.00,7.00]
Yu et al. (2022) (22)	68 (38–85)	71 (42–85)	86 (64.2%)	78 (58.6%)	91 (67.9%)	81 (60.9%)	46 (34.3%)	36 (27.0%)	5 (3–19)	6 (3–20)
Tao et al. (2025) (23)	69 (59–76)	69 (59–76)	254 (61.4%)	251 (60.1%)	279 (67.4%)	275 (65.8%)	145 (35.0%)	142 (34.0%)	6 (5–9)	6 (5–9)

### 3.3 Risk of bias and sensitivity analysis

**Figure 2:**
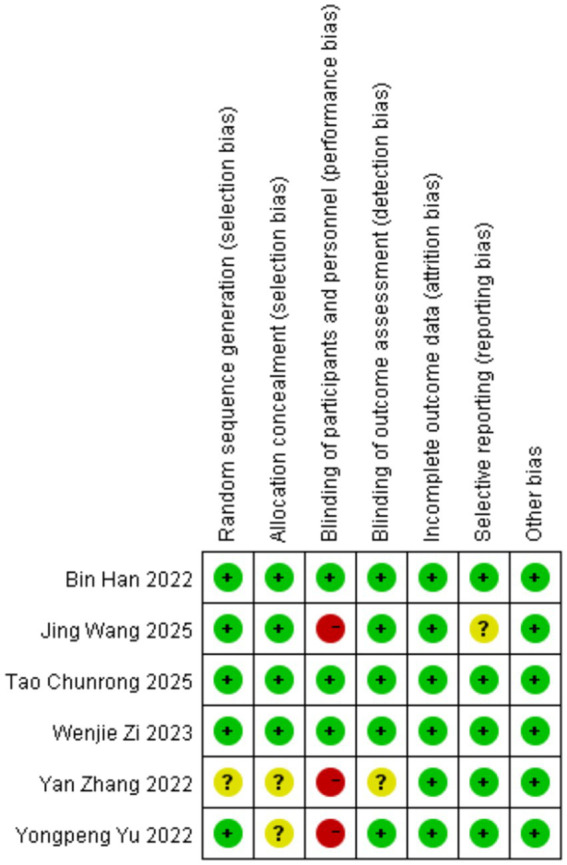
Risk of bias assessment for included randomized controlled trials (RoB 2.0).

Quality assessment was conducted using the ROBINS-I (Risk of Bias in Non-randomized Studies—of Interventions) tool for observational studies and the Cochrane Risk of Bias 2 (ROB 2) tool for RCTs. Among the nine included studies, six RCTs were rated as high quality, and three observational studies were considered of moderate quality (Figure 2). Sensitivity analyses demonstrated consistent results for both efficacy and safety outcomes. Publication bias was assessed via visual inspection of the funnel plot (Supplementary Figures S1–S3). As fewer than 10 studies were included, Egger’s test was not performed to assess publication bias. Visual inspection of the funnel plots indicated symmetry, suggesting no significant publication bias. The quality assessment of the included retrospective cohort studies is summarized in Table 3.

**Table 3:** Risk of bias using the ROBINS-I.

Study	D1	D2	D3	D4	D5	D6	D7	Overall
Xue et al. (2025) (21)	Moderate	Serious	Low	Moderate	Serious	Moderate	Low	Serious
Lin et al. (2017) (18)	Serious	Moderate	Low	Low	Low	Serious	Low	Serious
Liu et al. (2020) (16)	Moderate	Serious	Low	Moderate	Serious	Moderate	Low	Moderate

### 3.4 Efficacy outcomes

#### 3.4.1 90-day excellent functional outcome (mRS 0–1)

**Figure 3:**
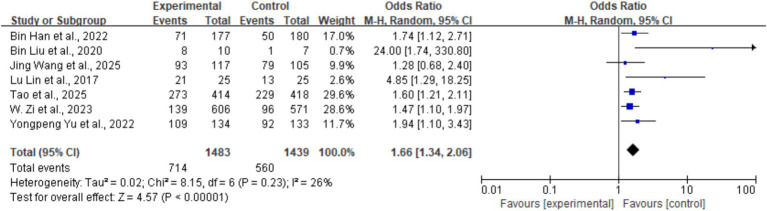
Forest plot for efficacy of tirofiban in obtaining excellent functional outcome (mRS 0–1).

Seven studies (five RCTs and two non-RCTs) reported this outcome, including 1,483 patients in the tirofiban group and 1,439 in the control group. The rate of excellent functional outcome was 48.1% (714/1,483) in the tirofiban group compared with 38.9% (560/1,439) in the control group, representing a statistically significant difference (OR = 1.66, 95% CI: 1.34–2.06, *p* < 0.001). The Mantel–Haenszel random-effects model was used for pooling. Heterogeneity analysis indicated low-to-moderate heterogeneity among the seven studies (*I^2^* = 26%, *p* = 0.23), which may be attributable to variations in study design, sample size, stroke severity, and treatment protocols. The forest plot for this outcome is shown in Figure 3.

#### 3.4.2 90-day favorable functional outcome (mRS 0–2)

**Figure 4:**
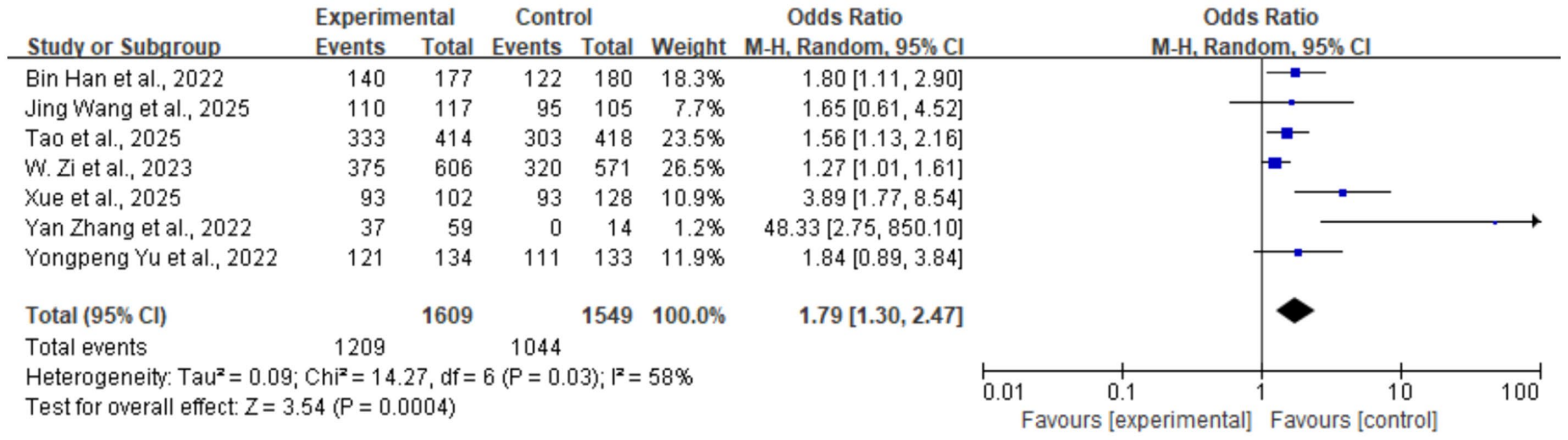
Forest plot of the effect of tirofiban on 90-day favorable functional outcome (mRS 0–2).

Seven studies (five RCTs and two non-RCTs) reported this outcome, encompassing a total of 3,158 patients (1,609 in the tirofiban group and 1,549 in the control group). The rate of favorable functional outcome was 75.1% (1,209/1,609) in the tirofiban group compared with 67.3% (1,044/1,549) in the control group. Pooled analysis indicated that tirofiban was associated with a significantly higher likelihood of achieving a favorable functional outcome (OR = 1.79, 95% CI: 1.30–2.47, *p* < 0.001). Moderate heterogeneity was observed among the seven studies (*I^2^* = 58%, *p* = 0.03). The forest plot for this outcome is shown in Figure 4.

### 3.5 Safety outcomes

#### 3.5.1 Symptomatic intracerebral hemorrhage (sICH)

**Figure 5:**
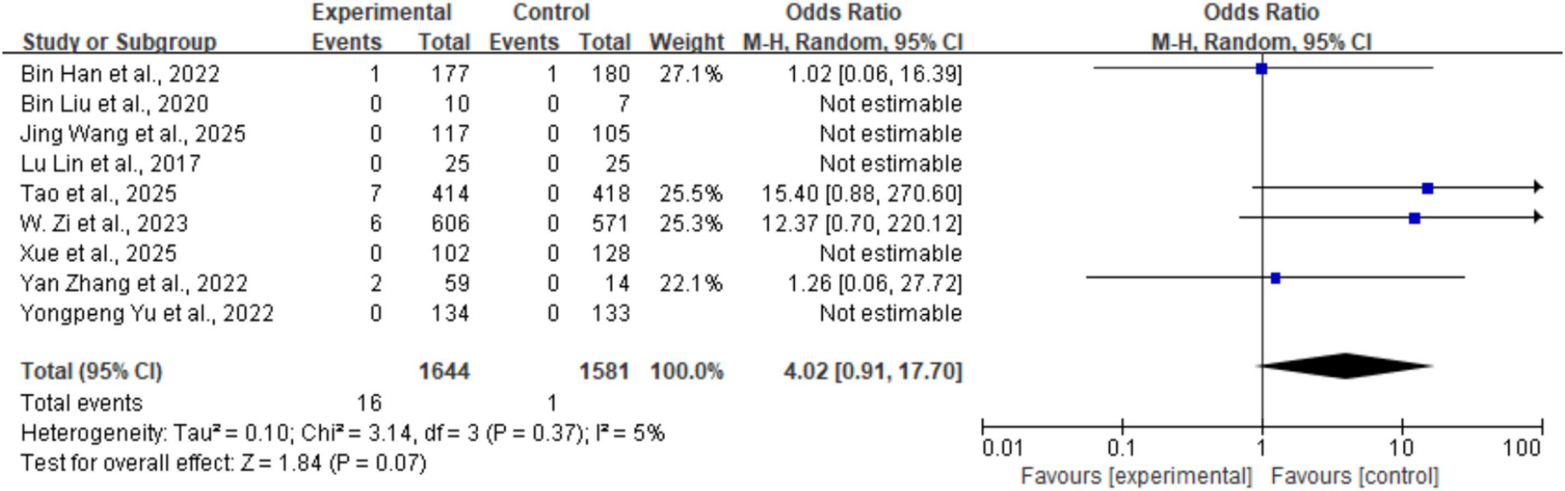
Forest plot of the effect of tirofiban on symptomatic intracerebral hemorrhage (sICH).

Across the nine studies, the overall incidence of symptomatic intracerebral hemorrhage (sICH) was 0.5% (17/3,225), with 16 cases occurring in the tirofiban group and 1 case in the control group. There was no statistically significant difference in sICH incidence between the tirofiban and control groups (OR = 4.02, 95% CI: 0.91–17.70, *p* = 0.07). Heterogeneity was low (*I^2^* = 5%, *p* = 0.37) (Figure 5).

#### 3.5.2 Any ICH

**Figure 6:**
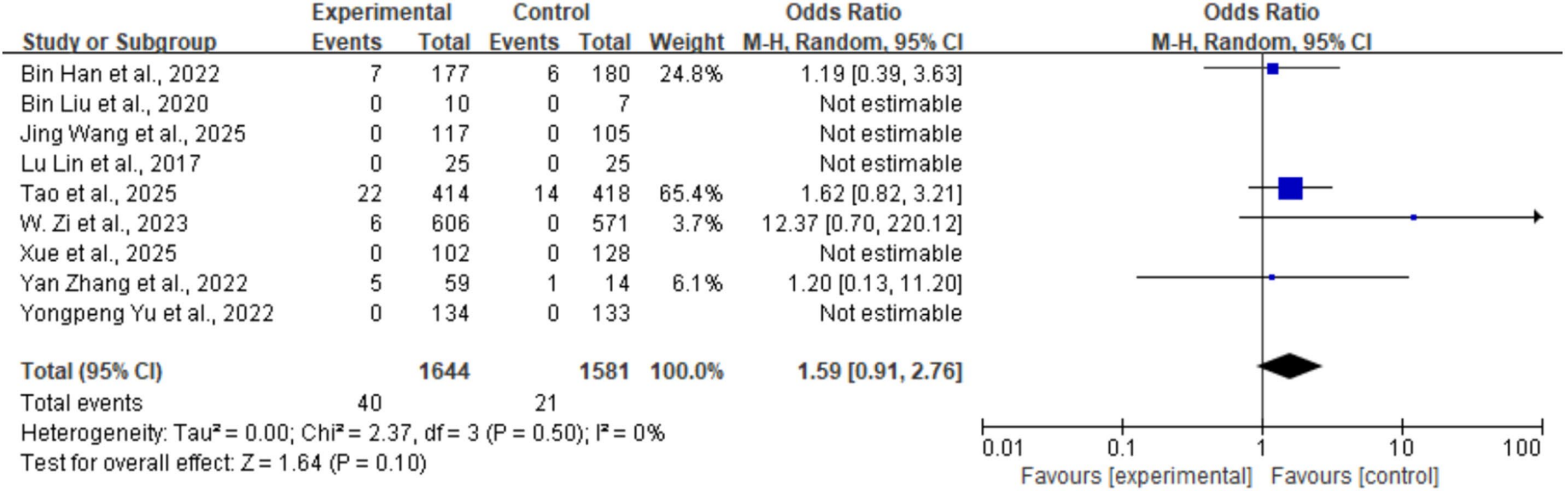
Forest plot of the effect of tirofiban on any intracerebral hemorrhage (any ICH).

The overall incidence of any intracerebral hemorrhage (ICH) was 1.8% (61/3,225), with 40 cases in the tirofiban group and 21 cases in the control group. There was no statistically significant difference in the incidence of any ICH between the two groups (OR = 1.59, 95% CI: 0.91–2.76, *p* = 0.10). No heterogeneity was observed across studies (*I^2^* = 0%, *p* = 0.50) (Figure 6).

#### 3.5.3 Peripheral bleeding

**Figure 7:**
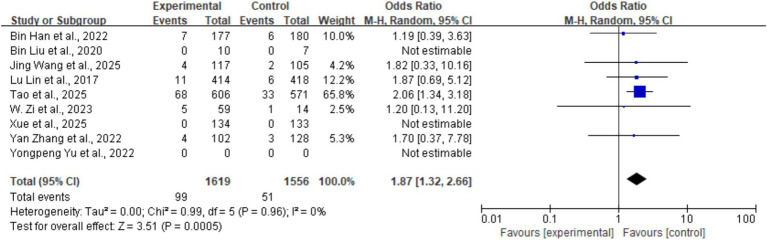
Forest plot of the effect of tirofiban on peripheral bleeding.

Peripheral bleeding was defined as hemorrhagic events outside the intracranial space, including gastrointestinal bleeding, hematuria, mucocutaneous bleeding, subcutaneous hematoma, and similar events. Eight studies reported this outcome, with an overall incidence of 4.7% (150/3,175): 6.1% (99/1,619) in the tirofiban group and 3.2% (51/1,556) in the control group. Pooled analysis showed that tirofiban was associated with a significantly higher risk of peripheral bleeding (OR = 1.87, 95% CI: 1.32–2.66, *p* < 0.001). No heterogeneity was observed among studies (*I^2^* = 0%, *p* = 0.96) (Figure 7).

#### 3.5.4 90-day mortality

**Figure 8:**
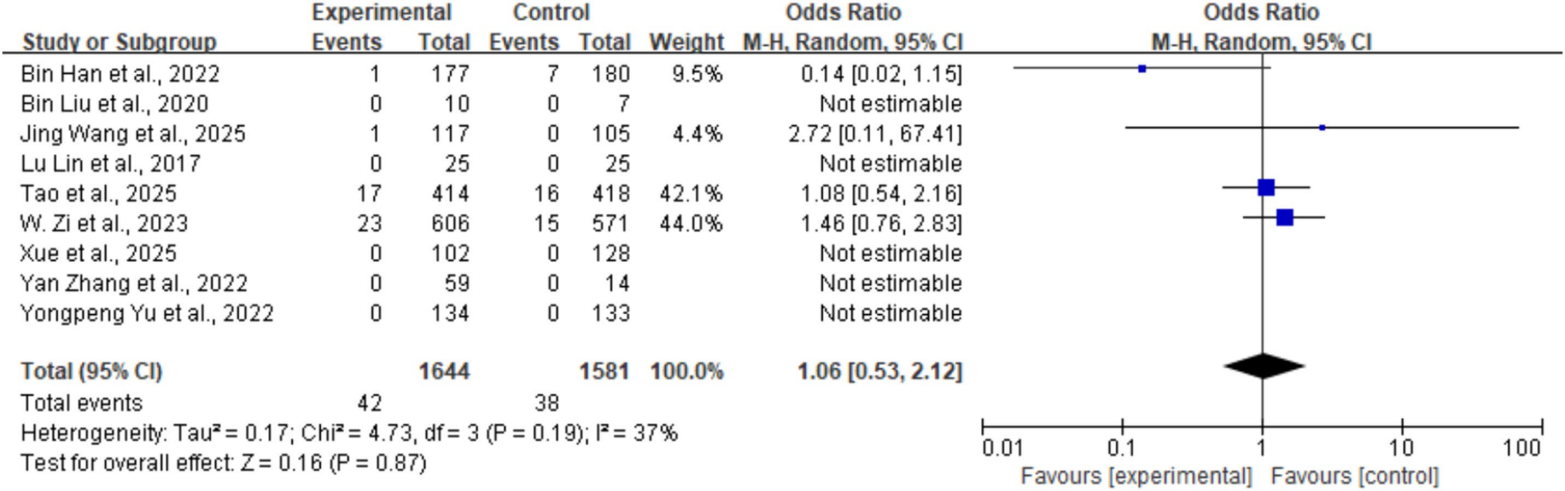
Forest plot of the effect of tirofiban on 90-day mortality.

The overall 90-day mortality was 1.9% (80/3,225), with 42 cases in the tirofiban group and 38 cases in the control group. There was no statistically significant difference in 90-day mortality between the two groups (OR = 1.06, 95% CI: 0.53–2.12, *p* = 0.87). Moderate heterogeneity was observed across studies (*I^2^* = 37%, *p* = 0.19) (Figure 8).

### 3.6 Subgroup analysis

#### 3.6.1 Subgroup analysis by IVT status

**Figure 9:**
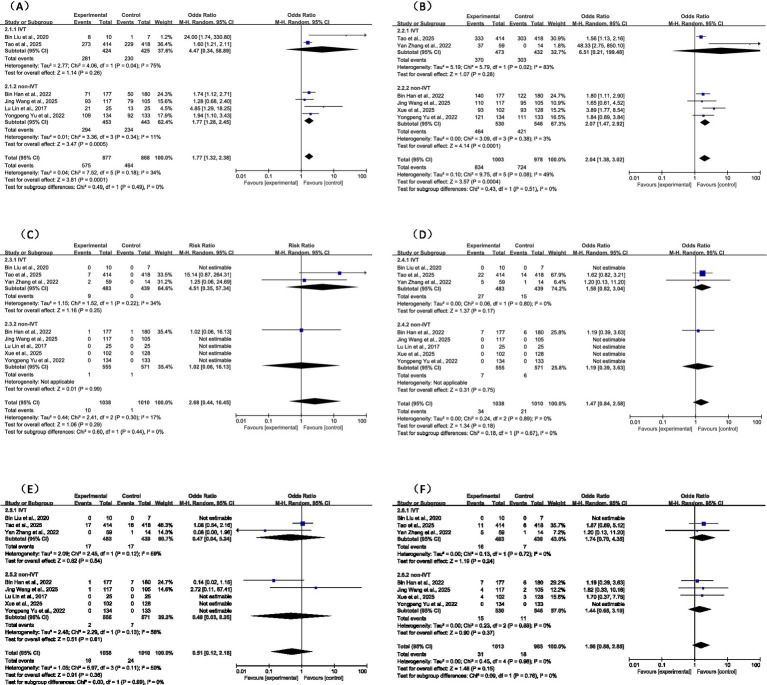
Subgroup analysis between IVT and non-IVT outcomes: **(A)** excellent functional outcome **(B)** favorable functional outcome **(C)** SICH **(D)** any ICH **(E)** morality **(F)** peripheral bleeding.

Subgroup analyses were conducted by stratifying patients according to prior IVT status (Figure 9). It is noteworthy that the interaction test yielded a non-significant result (*p* = 0.49), suggesting that the direction of the effect might be consistent across both IVT and non-IVT patients. However, statistical consistency does not equate to clinical efficacy in every subpopulation. In the non-IVT subgroup, tirofiban significantly increased the rates of 90-day excellent outcome (mRS 0–1: OR = 1.77, 95% CI: 1.28–2.45, *p* < 0.001) and favorable outcome (mRS 0–2: OR = 2.07, 95% CI: 1.47–2.92, *p* < 0.001), with low heterogeneity (*I^2^* ≤ 11%). Among patients who received IVT, tirofiban also suggested potential benefits (mRS 0–1: OR = 4.47; mRS 0–2: OR = 6.51), but these differences were not statistically significant (*p* > 0.05), likely due to high heterogeneity (*I^2^* ≥ 75%) from small sample sizes. No significant interaction was observed between IVT status and efficacy (all *p* > 0.40), supporting the overall consistent effectiveness of tirofiban.

Regarding safety outcomes, tirofiban showed a relatively low safety risk in both IVT and non-IVT patients. There were no statistically significant differences in sICH (OR = 2.68, *p* = 0.29), any ICH (OR = 1.56, *p* = 0.15), or 90-day mortality (OR = 1.47, *p* = 0.18) between tirofiban and control groups, regardless of IVT status. Importantly, tirofiban did not significantly increase the risk of peripheral bleeding in the subgroup analysis (OR = 0.51, *p* = 0.36). No significant moderating effect of IVT status on safety outcomes was detected (all *p* > 0.40, *I^2^* = 0%), indicating that tirofiban maintains a consistent safety profile in non-LVO/MVO stroke patients across different reperfusion contexts.

#### 3.6.2 Subgroup analysis by study design

**Figure 10:**
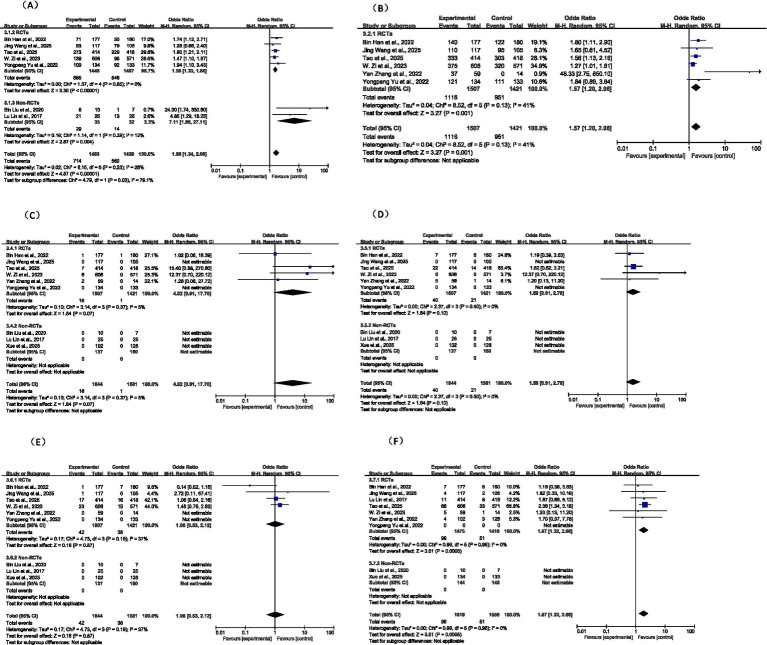
Subgroup analysis between RCT and non-RCT outcomes: **(A)** excellent functional outcome; **(B)** favorable functional outcome; **(C)** SICH; **(D)** any ICH; **(E)** morality; **(F)** peripheral bleeding.

Subgroup analyses were also performed by study design, separating RCTs and non-RCTs (Figure 10). In the RCT subgroup, tirofiban significantly increased the proportion of excellent outcomes (mRS 0–1: OR = 1.58, *p* < 0.001, *I^2^* = 0%) and favorable outcomes (mRS 0–2: OR = 1.57, *p* = 0.001, *I^2^* = 41%). Observational studies reported a larger apparent effect (mRS 0–1: OR = 7.11), leading to significant subgroup differences in the analysis of favorable outcomes (*p* = 0.03). These results indicate that while observational data may overestimate treatment effects, high-quality RCT evidence consistently supports the efficacy of tirofiban in improving functional recovery.

For safety outcomes, RCT-based evidence demonstrated favorable profiles. In the RCT subgroup, tirofiban did not significantly increase the risk of sICH (OR = 1.59, *p* = 0.10), any ICH (OR = 4.02, *p* = 0.07), or 90-day mortality (OR = 1.06, *p* = 0.87). However, a significantly higher risk of peripheral bleeding was observed (OR = 1.87, *p* < 0.001) without heterogeneity (*I^2^* = 0%). In the non-RCT subgroup, all four safety events were rare, preventing reliable estimation of effect sizes. Overall, no conflicting trends were observed between RCT and observational data within the evaluable outcomes, further confirming the safety of tirofiban in non-LVO/MVO stroke patients.

## 4 Discussion

This systematic review and meta-analysis evaluated the efficacy and safety of tirofiban in patients with non-LVO/MVO AIS to inform clinical practice. Over the past decade, treatment of AIS has advanced substantially, but research has primarily focused on endovascular therapy for LVO. Notably, 60–70% of clinical AIS cases are non-LVO. Previous studies have reported that although these patients often present with lower baseline NIHSS scores, 10–20% experience END (24), primarily due to in-situ thrombus progression, perforating artery ostial occlusion, and worsening microcirculatory perfusion in the ischemic penumbra (25).

The core findings of this meta-analysis demonstrate that tirofiban significantly improves 90-day functional outcomes, increasing the likelihood of an excellent outcome (mRS 0–1: OR = 1.77, 95% CI: 1.32–2.38) and a favorable outcome (mRS 0–2: OR = 2.04, 95% CI: 1.38–3.02) in non-LVO/MVO stroke patients. This highlights its role as an effective therapeutic option in this subgroup of stroke patients, where timely intervention is crucial for preventing neurological deterioration. Compared with studies on oral dual antiplatelet therapy (DAPT) (26, 27), tirofiban offers the advantages of rapid onset and reversible action as an intravenous glycoprotein IIb/IIIa receptor antagonist. Intravenous administration achieves peak plasma concentration quickly, with over 80% platelet receptor occupancy within minutes, and exhibits a fast dissociation rate. These pharmacological properties provide immediate and controllable antiplatelet activity in patients with unstable thrombus during the acute phase, offering timely protection for non-LVO patients in the unstable stage.

The highly consistent results observed in the RCT subgroup (*I^2^* = 0%) further support the biological efficacy of this rapid intervention in non-LVO/MVO patients. The consistency of the therapeutic efficacy observed in this study is largely attributable to the standardization of dosing regimens across the included trials. The majority of the studies (e.g., Zi et al., Tao et al.) followed a consistent protocol: a loading dose of 0.4 μg/(kg·min) for 30 min, followed by a maintenance infusion of 0.1 μg/(kg·min). This standardized dosage ensures rapid and sufficient inhibition of platelet glycoprotein IIb/IIIa receptors while mitigating the risk of uncontrollable hemorrhage associated with excessive doses, thereby providing a stable therapeutic window for patients with non-LVO/MVO stroke.

In non-LVO/MVO AIS, small vessel occlusion and BAD are important contributors to disability. Several reviews on BAD emphasize that its pathophysiology is primarily due to atherosclerotic plaque-induced luminal occlusion of perforating arteries rather than simple lipohyalinosis (28). Our analysis demonstrates that tirofiban provides robust benefits in small vessel stroke patients who are not candidates for EVT. Yamamoto et al. (29) suggested that intravenous antiplatelet therapy can effectively prevent “stepwise” neurological deterioration in BAD patients with progressive motor deficits (PMD). In contrast, conventional oral antiplatelet agents may inadequately inhibit dynamic thrombus evolution due to variable bioavailability. By pooling data from multiple RCTs, this study confirms that tirofiban may offer superior salvage or preventive effects for patients with microcirculatory failure caused by BAD or other small artery occlusions (SAO) by stabilizing thrombus formation on plaque surfaces.

Although the overall effects of tirofiban on functional outcomes are robust, moderate heterogeneity was observed (mRS 0–1: *I^2^* = 34%; mRS 0–2: *I^2^* = 49%), likely reflecting differences in study design, administration route, IVT status, timing of treatment initiation, and baseline patient characteristics. For instance, Yu et al. (22) extended the maintenance duration to 72–108 h, which may potentially further improved functional outcomes through more persistent microcirculatory protection, albeit at the expense of increased potential risks. Additionally, Xue et al. (21) explored a regimen involving an IA bolus followed by sequential IV infusion. Compared to pure IV administration, IA delivery allows the immediate achievement of drug concentrations at the thrombus site, which may explain the significant advantages in early recanalization rates observed in their study.

Specifically, in the IVT subgroup, although tirofiban showed potential benefits (mRS 0–1: OR = 4.47; mRS 0–2: OR = 6.51), these differences were not statistically significant (*p* > 0.05). This lack of statistical significance may be attributed to high heterogeneity (I^2^ ≥ 75%), which could reflect variations in study designs, patient populations, and the timing of treatment initiation, as well as small sample sizes in the IVT subgroup. Previous studies have suggested that in patients undergoing EVT, the adjunctive use of tirofiban following IVT showed a trend toward inferior clinical outcomes compared to the control group, accompanied by an elevated risk of hemorrhage (30). Consequently, the benefit–risk ratio of combined tirofiban administration appears suboptimal (31). This may be attributed to the fact that IVT, as a potent early-stage reperfusion therapy, facilitates thrombus dissolution through the conversion of plasminogen into plasmin, its intrinsic reperfusion efficacy is already robust in specific populations (32, 33). Under conditions of reduced thrombus burden post-thrombolysis, the capacity for further antiplatelet therapy to yield additional clinical benefits may be constrained. Therefore, when tirofiban is superimposed on IVT, its marginal benefit is effectively diluted.

In contrast, the RCT subgroup demonstrated high consistency (*I^2^* = 0%), providing high-quality evidence to support the clinical use of tirofiban. Observational studies suggested a larger apparent benefit (OR = 7.11), but their effect sizes were significantly higher than those of RCTs (*p* = 0.03), likely due to smaller sample sizes and heterogeneous inclusion criteria across centers.

A key clinical concern is whether the immediate combination of tirofiban with IVT increases the risk of hemorrhagic transformation (HT). The overall recanalization rate with IVT using rt-PA is approximately 46, and 14–34% of patients experience reocclusion (34). One important mechanism of reocclusion is IVT-induced hypercoagulability and platelet activation. Accordingly, combining thrombolysis with antiplatelet therapy may help reduce reocclusion. However, previous clinical studies (35, 36) have reported that antiplatelet therapy within 24 h of thrombolysis significantly increases the risk of intracranial hemorrhage without improving functional outcomes. In our safety analysis, tirofiban did not significantly increase the risk of sICH after IVT (OR = 2.68, *p* = 0.29). Jang et al. (37) noted that high-dose intravenous tirofiban may be associated with a higher bleeding risk, whereas low-dose or intra-arterial administration carries a lower risk. Recent studies have largely adopted a no-bolus maintenance infusion strategy after thrombolysis, which avoids excessive platelet inhibition while maintaining microcirculatory patency. Our findings support a synergistic effect between thrombolytic agents and low-dose tirofiban in non-LVO AIS: thrombolytics degrade existing fibrin, while tirofiban prevents new platelet aggregation without disrupting formed hemostatic plugs. Although the RCT subgroup showed an increased risk of peripheral bleeding, these events were predominantly minor and non-fatal, yielding a balanced risk–benefit profile. Tirofiban may therefore serve as an effective adjunctive therapy for non-LVO/MVO patients who exhibit poor neurological improvement after thrombolysis or are ineligible for thrombolysis, facilitating functional recovery and a return to high-quality daily life.

## 5 Limitations

This meta-analysis has several limitations. First, all included studies were conducted in China, and the generalizability of these findings to non-LVO/MVO stroke patients of other ethnicities remains uncertain. Second, protocols for tirofiban loading doses varied across studies. For example, Tao et al. (23) used a low-dose protocol to maximize post-thrombolysis safety, whereas Zi et al. (19) administered a full loading dose. Such clinical heterogeneity may affect the assessment of safety outcomes. Third, safety outcomes were primarily derived from large RCTs, while smaller observational studies contributed little to the safety analysis due to zero-event reporting. Finally, although sensitivity analyses and funnel plots suggested stable results and a low risk of publication bias, the small number of studies included for each outcome may limit statistical power. Some studies were non-randomized, introducing potential selection bias and incomplete adjustment for confounding factors.

## Data Availability

The original contributions presented in the study are included in the article/Supplementary material, further inquiries can be directed to the corresponding authors.
