# Supplementary material for: Efficacy and safety of tirofiban for acute ischemic stroke without large and medium vessel occlusion: a systematic review and meta-analysis
Source: Front Neurol. 2026 May 4;17:1823316. doi: 10.3389/fneur.2026.1823316 (PMC13181925; doi:10.3389/fneur.2026.1823316)
Supplement: Supplementary file 1 [file Data_Sheet_1.docx]

Supplementary Material

Efficacy and Safety of Tirofiban for Acute Ischemic Stroke Without Large and Medium Vessel Occlusion: A Systematic Review and Meta - Analysis

**Supplemental Material**

This file includes:

**Figures S1** Funnel plots of the main outcome

**Figures S2** Funnel plots of subgroup analysis between IVT and non-IVT outcomes

**Figures S3** Funnel plots of subgroup analysis between RCT and non-RCT outcomes

Figure S 1 | Funnel plots: (A) 90-Day Excellent Functional Outcome (mRS 0-1). (B) 90-Day Favorable Functional Outcome (mRS 0-2). (C) sICH. (D) Any ICH. (E) Peripheral Bleeding. (F) 90-Day Mortality.

Figure S 2 | Funnel plots of subgroup analysis between IVT and non-IVT outcomes: (A) 90-Day Excellent Functional Outcome (mRS 0-1). (B) 90-Day Favorable Functional Outcome (mRS 0-2). (C) sICH. (D) Any ICH. (E) Peripheral Bleeding. (F) 90-Day Mortality.

Figure S 3 | Funnel plots of subgroup analysis between RCT and non-RCT outcomes: (A) 90-Day Excellent Functional Outcome (mRS 0-1). (B) 90-Day Favorable Functional Outcome (mRS 0-2). (C) sICH. (D) Any ICH. (E) Peripheral Bleeding. (F) 90-Day Mortality.
